# *Haootia quadriformis* n. gen., n. sp., interpreted as a muscular cnidarian impression from the Late Ediacaran period (approx. 560 Ma)

**DOI:** 10.1098/rspb.2014.1202

**Published:** 2014-10-22

**Authors:** Alexander G. Liu, Jack J. Matthews, Latha R. Menon, Duncan McIlroy, Martin D. Brasier

**Affiliations:** 1 Department of Earth Sciences, University of Cambridge, Downing Street, Cambridge CB2 3EQ, UK; 2 Department of Earth Sciences, University of Oxford, South Parks Road, Oxford OX1 3AN, UK; 3 Department of Earth Sciences, Memorial University of Newfoundland, 300 Prince Philip Drive, St John's, Newfoundland and Labrador, Canada A1B 3X5

**Keywords:** Ediacaran, metazoan, Newfoundland, Cnidaria, muscle

## Abstract

Muscle tissue is a fundamentally eumetazoan attribute. The oldest evidence for fossilized muscular tissue before the Early Cambrian has hitherto remained moot, being reliant upon indirect evidence in the form of Late Ediacaran ichnofossils. We here report a candidate muscle-bearing organism, *Haootia quadriformis* n. gen., n. sp., from approximately 560 Ma strata in Newfoundland, Canada. This taxon exhibits sediment moulds of twisted, superimposed fibrous bundles arranged quadrilaterally, extending into four prominent bifurcating corner branches. *Haootia* is distinct from all previously published contemporaneous Ediacaran macrofossils in its symmetrically fibrous, rather than frondose, architecture. Its bundled fibres, morphology, and taphonomy compare well with the muscle fibres of fossil and extant Cnidaria, particularly the benthic Staurozoa. *Haootia quadriformis* thus potentially provides the earliest body fossil evidence for both metazoan musculature, and for Eumetazoa, in the geological record.

## 1. Introduction

Sediments of Late Ediacaran age (approx. 580–541 Ma) record the fossilized remains of a diverse global assemblage of soft-bodied macro-organisms. The biological affinities of these Late Ediacaran macrofossils remain the subject of considerable debate (summarized in [1]). Following their initial discovery, Ediacaran soft-bodied organisms were commonly assigned to metazoan groups (e.g. [2], or the classification tables in [3], pp. 240–242). However, the revolution in Ediacaran thinking brought about by the Vendobiont hypothesis of Seilacher [4] led to reconsideration of many of those assignments. Recent years have witnessed a trend towards interpreting individual taxa as candidate stem- and crown-group metazoans. Described with varying degrees of confidence, these currently include potential sponges [5–8], anthozoan, hydrozoan and scyphozoan cnidarians [9–11], ctenophores [12], placozoans [13], early molluscs ([14]; though see [15]) and even ascidian chordates [16]. These fossils are largely found in successions of approximately 555–541 Ma, in South China, Brazil, the White Sea region of Russia, Namibia and the Flinders Ranges of South Australia [17,18]. Further evidence for the presence of metazoans in the Late Ediacaran period, and indirectly for muscular tissue, comes from simple, putatively bilaterian, surface trace fossils from the previously mentioned localities [19–21], horizontal surface traces with crescentic internal divisions made by motile, muscular organisms [22,23] approximately 565 Ma [24], and vertical equilibration traces from Newfoundland [23]. Prior to 565 Ma, the potential fossil record of animals is restricted to claims for biomarkers (e.g. demosponge steranes of more than 635 Ma [25]; though see [26]); various specimens interpreted as possible sponges from the Early and Middle Neoproterozoic ([27–29]; though see [8]); and traces of contested age and origin [30–32]. The absence of clear metazoan body fossils until the latest Ediacaran Period renders these earliest reports open to debate. Independent estimates for the first appearance of animals in the Neoproterozoic vary widely, but recent molecular phylogenetic studies predict that most stem-group divergences between extant metazoan phyla occurred within the Cryogenian and Ediacaran Periods [33].

**Figure 1.**
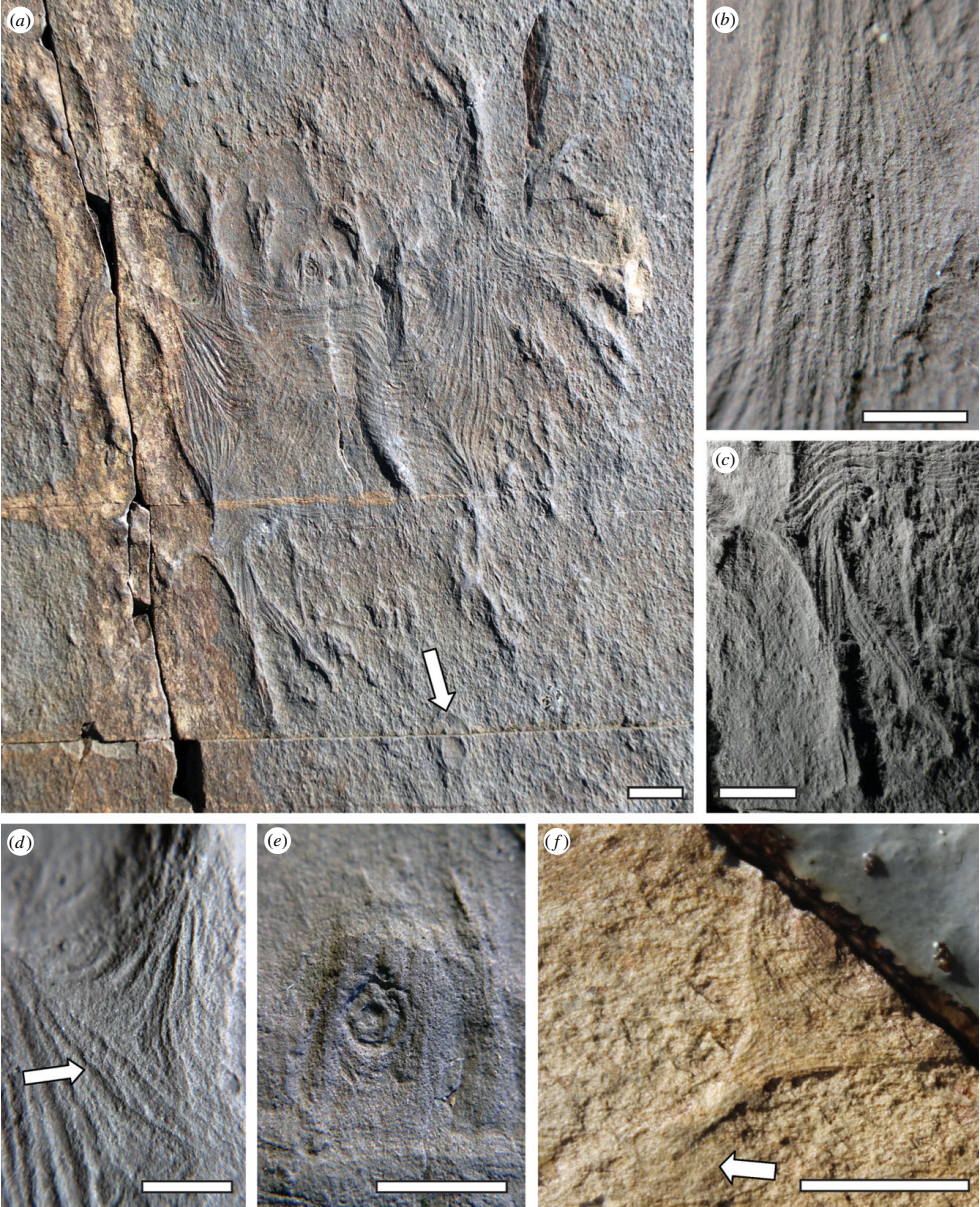
*Haootia quadriformis* n. gen., n. sp., lower Fermeuse Formation of Back Cove, Bonavista Peninsula, Newfoundland*.* (*a*) *Haootia quadriformis* holotype specimen. Note the negative-relief central disc, interpreted as a holdfast, and the broadly bilaterally symmetrical bundles of linear ridges, extending into discrete bifurcating branches. Inferred current direction indicated by the arrow. (*b*) Fibres running along the right-hand margin of *Haootia*; each fibre is composed of finer, thinner fibres. (*c*) Bottom left corner of *Haootia*, detailing the connection between a primary bifurcating branch and the main body. Note the twisted fibres along the branch. (*d*) Pinching, bundling and superposition of fibres at the base of a subsidiary branch. (*e*) The small circular depression at the centre of the disc, showing mantling parallel fibres forming the base of a short stalk that connects the disc to the body. (*f*) Incomplete paratype specimen of *H. quadriformis*, from the Trepassey Formation of Burnt Point, Bonavista Peninsula. This specimen is preserved on its side, but clearly displays fibres extending up its stem and around the body. A small partially buried holdfast disc is arrowed. Scales bars (*a*,*c*,*f*), 10 mm; (*b*,*d*,*e*), 5 mm.

Newfoundland, in eastern Canada, contains some of the oldest non-algal Ediacaran macrofossil assemblages, dated to approximately 579–560 Ma [34]. Although ichnological evidence for the presence of metazoans in assemblages of this age has been reported [22,23,35], metazoan body plans have yet to be convincingly demonstrated. We here report *Haootia quadriformis* n. gen., n. sp. (figure 1) from the lower Fermeuse Formation of the Bonavista Peninsula of Newfoundland (approx. 560 Ma; electronic supplementary material, figure S1 and text S1). This organism exhibits structures wholly consistent with collagenous musculature, in the form of twisted and superimposed fibrous bundles arranged in a quadrilaterally symmetrical pattern.

## 2. Systematic palaeontology

Phylum CNIDARIA Hatschek, 1888 [36]

Genus HAOOTIA gen. nov.

*Derivation of name*. From the Beothuk (language of the indigenous population of Newfoundland) term *Haoot*, meaning *demon*, describing the striking appearance of the holotype.

*Type species. Haootia quadriformis* n. gen., n. sp.

*Diagnosis* (of genus). Soft-bodied, quadrilaterally symmetrical organism possessing a smooth discoidal structure connected by a relatively short stem to a quadrate body comprising numerous regularly aligned linear fibres. The fibres extend laterally across the body, linking adjacent corners. Converging fibres extend beyond each corner to form an elongate branch, which divides dichotomously to form smaller, distally tapering sub-branches. Smaller branches also emanate from the lateral margins of the quadrate body, and these too branch dichotomously.

*Haootia quadriformis* sp. nov.

*Derivation of name.* From the Latin *quadri* (fourfold), and *formis* (form), relating to the quadrilateral symmetry of the organism's body.

*Holotype.* The original specimen, discovered by M.D.B. in 2008, remains uncollected in the field according to provincial law in Newfoundland. A plastotype is held within the collections of the Oxford University Museum of Natural History, specimen OUM ÁT.424/p.

*Horizon and locality.* From the lower part of the Late Ediacaran Fermeuse Formation, St John's Group [37]. The specimen resides within a turbiditic marine succession (electronic supplementary material, text S1 and figure S2) on the north shore of Back Cove, roughly 1.8 km NNW of the town of Melrose, Bonavista Peninsula, Newfoundland, Canada (electronic supplementary material, figure S1).

*Diagnosis.* As per the genus.

*Remarks. Haootia quadriformis* n. gen., n. sp. is known from the holotype specimen, and one additional incomplete specimen from the Trepassey Formation of Burnt Point, Bonavista Peninsula (figure 1*f*; electronic supplementary material, figures S1 and S5; designated the paratype). The smaller paratype specimen has been preserved in lateral view and displays an anchoring support structure, lineated stem and a furrowed body with apparent branches (figure 1*f*; electronic supplementary material, figure S5).

*Description.* The non-retrodeformed holotype bears a discoidal structure 56 × 37 mm in diameter, preserved in negative epirelief. The disc interior is smooth, apart from faint concentric ridges at its outer margin (figure 1*a*), and a small slightly raised central structure of 9 mm diameter with several tight concentric rings (figure 1*e*). This central structure appears to form the attachment point for a short 7-mm wide, lineated stalk-like structure, 32 mm in length, which extends to the centre of the quadrate body (figure 1*a*). The body is preserved as a rectangular sheet 49 × 72 mm in dimension, characterized by well-defined positive epirelief linear ridges (fibres) that are 100–600 µm wide and have peaks spaced 200 µm–1 mm apart. Individual fibres are finely lineated, exhibiting a structure composed of bundles of parallel strands (figure 1*a*,*b*). In places, these strands split and then re-join (figure 1*b*). At the four corners of the body, the fibres converge to form bundles that progress distally into elongate extensions, here termed branches (figure 1*c*). Each of the four corner branches bifurcates up to three times, and taper towards their distal end, with those fibres that persist distally decreasing in number after each successive branching point (figure 1*a*,*c*). Branches were originally flexible, as demonstrated by 180° changes in direction of some examples to face the predominant flow direction (as inferred from alignment of nearby unipolar rangeomorphs and *Charniodiscus* specimens; figure 1*a*), and by their apparent ability to become twisted and rotated (figure 1*c*). Location of the bulk of the organism down-current of the circular disc in both known specimens is consistent with entrainment by a flow on the seafloor prior to burial (figure 1*a*,*f*; electronic supplementary material, figure S5).

Along the margins of the body sheet, between the four corners, further smaller bundles of linear fibres converge to form small branches that divide dichotomously. Additionally, along the two shorter edges of the compacted body, linear fibres running from the adjacent corners combine to form bundles that bulge in the middle (figure 1*a*). By contrast, along the two longer edges, the fibres are less obviously clustered into discrete structures, and continue broadly parallel to one another.

A prominent linear structure preserved in positive epirelief runs up the centre-right of the impression, and the fibres of the surface of the body appear to drape over it (figure 1*a*). The narrow morphology of this structure and its similar topographic relief to the branches leads us to suggest that it reflects a primary branch from the lower right corner (as seen in figure 1*a*), folded beneath the body at the time of burial.

**Figure 2.**
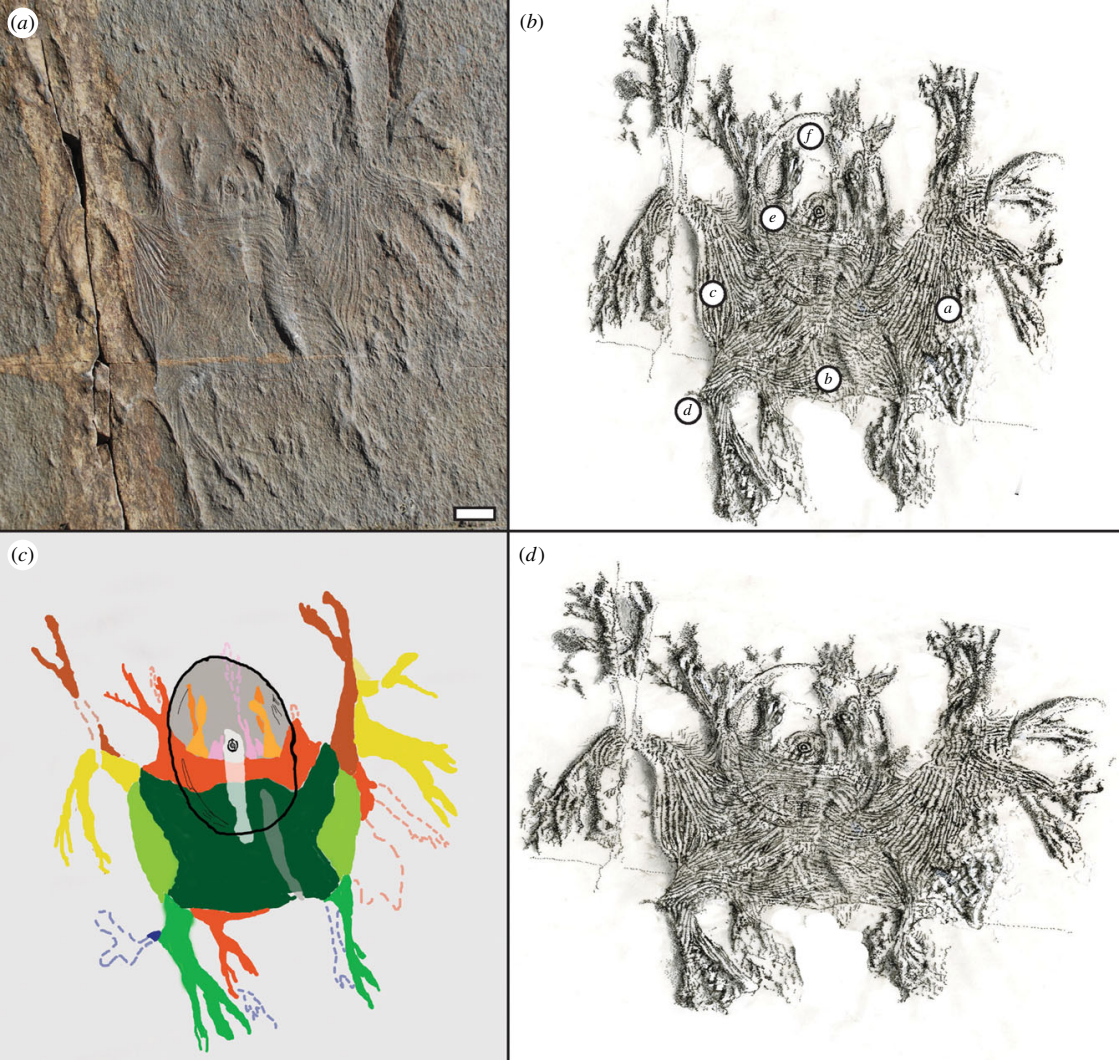
Digitized images of *H. quadriformis* n. gen., n. sp., emphasizing the convergence of fibrous linear features at the corners of the body, and the symmetry of the fossil. (*a*) Photograph of the holotype as it appears *in situ*. (*b*) Interpretive sketch of the non-retrodeformed specimen. Labels indicate: (*a*) muscle bundles, (*b*) expanded bundles, (*c*) ‘contracted’ bundles, (*d*) twisting fibres, (*e*) superimposed fibres and (*f*) disc. (*c*) Digitized overlay of the fossil. Symmetrical regions of the organism are colour coded. Note the thick bulging of fibres (indicating muscle contraction?) along short axes of the sheet (light green). (*d*) As in (*b*), but the image has been corrected to account for tectonic deformation on the surface by compressing the disc into a perfectly circular structure (cf. [38], though see [39]). Scale bar, 10 mm.

**Figure 3.**
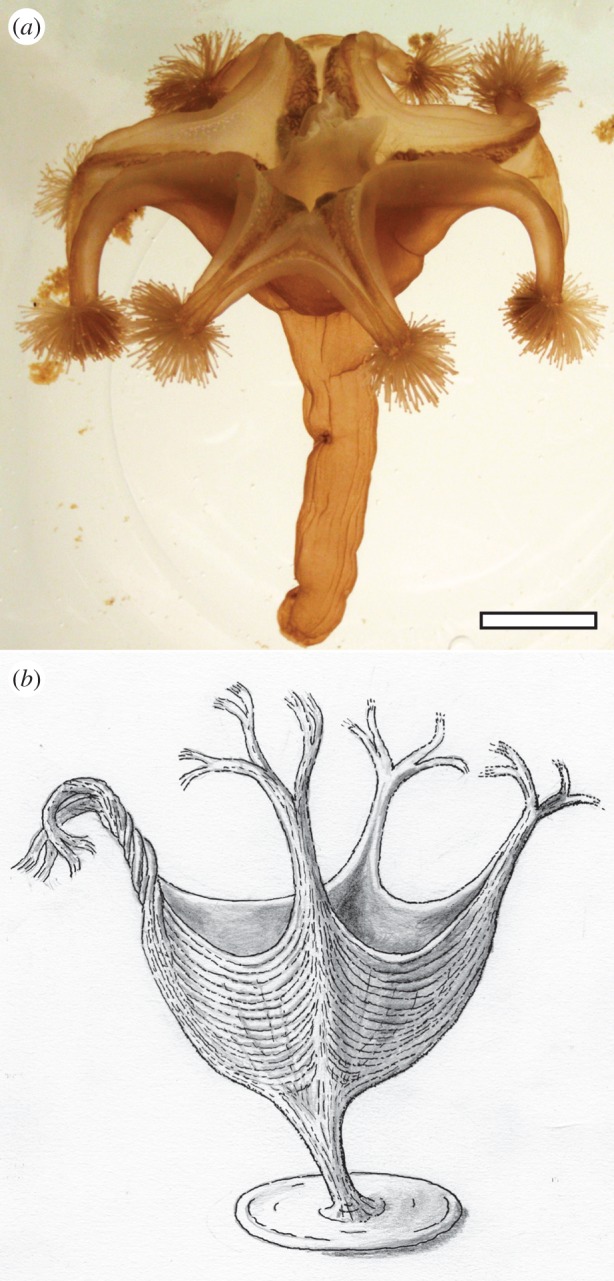
(*a*) The extant staurozoan *Lucernaria quadricornis*, exhibiting a body plan similar to that hypothesized for *H. quadriformis* n. gen., n. sp*.* The Staurozoa are known from a range of marine depositional environments and water depths [40]. (*b*) Artistic reconstruction of *H. quadriformis*. Scale bars, 10 mm.

*Discussion.* *Haootia quadriformis* displays several unique morphological traits, the most striking of which is an apparently symmetrical, fibrous body with regularly arranged branches (figure 2*b*). The superficial impression of bilateral symmetry in the holotype (figure 2*c*) was arguably brought about by oblique collapse and differential contraction of the body. Biostratinomic distortion is further enhanced by tectonic stretching. We thus infer that the original body was quadrilaterally symmetrical in life (figures 2*d* and 3*b*), and we suggest that the bedding plane relationships of the holotype specimen indicate composite preservation of a mould of the base of the anchoring adhesive disc, and the upper surface and internal structure of the body. The apparent draping of the quadrate body over the disc edge implies that the body lay above both the disc and stem on the seafloor at the time of burial (figure 1*a*). On the basis of the position of the disc upstream of the quadrate body, we infer that the disc was a tethering structure similar to those of associated frondose taxa (e.g. electronic supplementary material, figure S3*a*–*c*), and that *Haootia* was epibenthic.

The complex structure of *H. quadriformis*, with prominent bundles of fibres showing consistent directional changes within a discrete sheet-like structure, is not readily explained by tectonic or sedimentological processes. Unusual environmental taphonomic conditions can also be ruled out, because neighbouring specimens of recognizable macrofossil taxa on the bedding planes (e.g. figure 1*a*) do not differ in preservation or appearance from those found abundantly throughout the region. All other fossil impressions on these surfaces (electronic supplementary material, figure S3) lack fibrous structures of the kind described here.

## 3. Is this a known ediacaran macrofossil taxon?

Whereas typical frondose Ediacaran taxa possess either leaf-like morphologies or some evidence for alternating rangeomorph branching elements [41,42], such features are lacking in *Haootia. Primocandelabrum* sp. [37] (electronic supplementary material, figure S6*d*), a superficially similar contemporaneous rangeomorph bearing multiple branches attached by a stem to a disc, can be distinguished by its lack of quadrilateral symmetry, and its rangeomorph branching. Furthermore, in rare specimens where longitudinal ridges are preserved along the length of a *Primocandelabrum* [43], such ridges are wider, more broadly spaced and less regular in arrangement than those seen in *Haootia*. The disc in the holotype *Haootia* specimen also differs distinctly from others found on the same surface, being smoother, with lower topographic relief (figure 1*a*) and fewer concentric rings (electronic supplementary material, figure S3).

Examples of putative tissue differentiation in Ediacaran macrofossils have typically proved controversial. Structures interpreted as external sheaths and membranes have been described in *Pteridinium* and *Rangea* from Namibia [44,45], and in rare rangeomorphs from Newfoundland [46], although the latter examples likely have a sedimentological origin [47]. Such claimed sheaths are typically smooth and lack the fibrous character of *Haootia*. The internal anatomy of other Ediacaran macrofossils is largely inferred from composite impressions explained by biostratinomic collapse of tissues (e.g. [48], fig. 2), or from three-dimensional specimens in-filled by sediment (e.g. [49,50]). However, such typically lobate structures do not exhibit the wavy fibrous symmetry of *H. quadriformis*. Whereas the linear fibrous construction of the alga *Flabellophyton* from South China and Australia [51] shows some similarity with fibres of *Haootia*, those fossils lack a large holdfast, a stem-mounted body or quadrilateral symmetry. It could be argued that the linear fibres in *Haootia* result from the deformation or twisting of a non-muscular integument, but that cannot explain their presence across the whole body, their multi-directionality or their symmetry. Rough comparison may be made with the ‘crumpled’ margins of *Karakhtia* from the White Sea [52], but the folds in *Karakhtia* are irregular in shape and direction, radiate from the centre of the organism to the outer margin, and become more finely spaced towards the specimen edges. Differences are also apparent when considering linear features associated with ‘mop’ structures in Australia. ‘Mop’ plausibly results when a disc, embedded in a microbial mat, has been dragged by unidirectional currents [53] to produce unidirectional or evenly radiating marks. By contrast, *Haootia* fibres form bands that are multidirectional, often running parallel to the margins of the impression and appearing to converge with neighbouring fibres (figure 1*a*). Longitudinal furrows are known within ribbon-like *Harlaniella* [54]. Such linear features demonstrate how individual Ediacaran taxa can exhibit a variety of putative internal morphologies as a result of differential taphonomic processes. Such features will also require explanation, but on the available evidence, we do not consider *Haootia* to represent a taphonomic variant of any currently known Ediacaran taxon. Contemporaneous microbial fabrics can exhibit linear striated morphologies (e.g. Arumberia [55]), but are not typically localized in their occurrence, do not possess a sharp boundary to the impression, and are not known to form symmetrically arranged bifurcating structures.

## 4. Metazoan affinities?

*Haootia*'s size and complex, regular morphology demand consideration of metazoan affinities. Its symmetry and the lack of evidence for pores or spicules argue against Porifera (following [8]). The presence of numerous branches, absence of comb rows and inferred benthic mode of life likewise make comparison with Ctenophora problematic. Possession of quadrilateral structure, a central radial disc and fibrous soft tissues, clearly invite comparison with living and fossil Cnidaria.

Although the extant phylum Cnidaria includes morphologically and genetically disparate taxa [56,57], their molecular phylogeny confirms a basal position within the Eumetazoa [58]. Cnidarians are classically united by the possession of cnidocytes, diploblastic construction and radial symmetry, but suggestions of a wider variety of symmetry states (e.g. [59–61]) are supported by genetic arguments for the presence of bilateral symmetry in the eumetazoan common ancestor [62], and the presence of a mesoderm-like layer has been recognized in some cnidarian taxa (cf. [63]; electronic supplementary material, text S2).

The bundles of fibrous ridges within the body of *Haootia* compare favourably in size, order and arrangement to the preserved muscular tissue of modern cnidarians. Cnidarians can possess smooth and/or striated muscular tissue [63,64] (electronic supplementary material, text S2), both of which can form fibrous bundles arranged in a similar manner to those in *Haootia* [65] (figure 3*a*; electronic supplementary material, figure S6). Rare fossil examples of cnidarian muscular tissue (e.g. [66–68]) typically comprise impressions of regularly arranged ridges (e.g. [67], p. 63, fig. 55). These are best known in fossil scyphozoan medusae, where coronal and radial muscles of the sub-umbrella are often grouped into bundles (e.g. [69]) and are preserved as casts and moulds in a taphonomic style similar to that seen in the Ediacaran siliciclastic settings of Newfoundland [70]. The morphology of soft-bodied fossil cnidarians is typically influenced by muscle contraction at the time of burial [67]. Twisting and overlapping of fossil medusa tentacles [71] also compare closely with *Haootia*'s flexible branches. Phalloidin fluorescence reveals that the 1–2.5 µm-width smooth muscle fibres in the extant parasitic hydrozoan *Polypodium hydriforme* run longitudinally up the length of the tentacles [65] in an arrangement strikingly similar to individual fibres in *H. quadriformis*. Furthermore, the junction between muscles in the tentacles and those in the body of *P. hydriforme* produces a similar ‘truncated’ surface to the ridges observed in *Haootia* (figure 1*d*; [65], fig. 4*a*), and individual fibres can also split and/or join one another. These morphological and structural similarities lead us to the conclusion that the fibrous structures preserved within *Haootia* may well represent the soft tissue impressions of cnidarian musculature. If so, this specimen significantly pre-dates previously documented preserved muscular tissues, the oldest of which are Early Cambrian in age [72,73].

Striated muscle fibres have been demonstrated to be present in the cubozoan *Tripedalia cystophora* ([74], fig. 5), and although individual fibres are of smaller magnitude than those seen in *H. quadriformis*, they are nevertheless very similar in gross morphology. Smooth muscle has also been observed to form macroscopic fibrous bundles within the tentacles of several scyphozoans [63] and cubozoans [74,75]. Distinguishing between bundles of smooth and striated muscle cells in the fossil record is not likely to be possible when only soft tissue impressions are available for study. In the living actinian *Metridium*, the better-developed (smooth) longitudinal muscles are notably found in the ectoderm of the tentacles, with circular muscles located in the endoderm ([76], p. 79; *contra* [77]). This differentiation of muscle groups within different tissues may explain why we only see longitudinal ridges along the branches of *Haootia*, with no clear evidence for circular bands.

The preservation of muscular tissue in the Phanerozoic is uncommon and is typically restricted to Konservat Lagerstätten [78]. In many cases, particularly involving arthropod and vertebrate muscle, preservation takes place via authigenic replacement of muscular tissues by calcium phosphate or clay minerals [79], or via sulfurization of organic matter [68]. In the Ediacaran, taphonomic processes were significantly different, and soft tissue preservation was commonly facilitated by the early diagenetic, microbially induced casting of fossil exteriors in framboidal pyrite [47,80] or by rapid burial beneath volcanic ash [81]. Such mouldic preservation is unusual in the Phanerozoic, but has been documented to preserve cnidarians (and significantly impressions of their muscular tissue) at several localities [71].

An important consideration is explaining how internal muscle tissues are preserved in this manner, when in other Ediacaran macrofossils we typically only see external morphology. In taphonomic experiments involving modern hydrozoans and scyphozoans, impressions of muscular tissues were not preserved [82,83]. However, the absence of microbial mats on the experimental surfaces [82], and the desiccation of specimens [83], precludes direct comparison between those studies and Ediacaran taphonomic conditions. We suggest that rapid degradation of an external integument in *Haootia* (such as the epidermis, less than 50 µm thick in some modern cnidarians [84]) upon death and burial exposed the relatively more robust muscular tissues and permitted them to be cast in the same manner as contemporaneous Ediacaran macrofossils.

We infer that the muscle-like fibres seen in *Haootia* likely facilitated extension and retraction of branches for gathering food, as with the tentacles of modern cnidarian polyps. We see neither a distinct mouth-like structure nor a gastro-vascular cavity, so their presence must be inferred at the centre of the quadrilateral body. Similarly, structures similar to canals or mesenteries are not clearly distinguishable. Interpretation of the disc as a benthic holdfast then implies a polyp-like organism, with a gross body-plan most similar to that of living staurozoans (e.g. figure 3). The fibres within *Haootia* are consistent with the positioning of muscular fibres in the calyx of modern Staurozoa [85] (figure 3*a*), being longitudinal within the stalk and branches of the specimen but mainly positioned laterally (i.e. parallel to the margins in a manner analogous to coronal musculature in modern forms [84]) in the body. However, the additional marginal branches in *Haootia* are unlike anything seen in staurozoans, which typically possess only eight arms. *Haootia* also lacks fossilized evidence for morphological features such as anchors, gonads, nematocyst clusters or characteristic tissue structures observed in histological sections through modern Staurozoa (e.g. ref. [84]). As *Haootia* is also considerably larger than most extant Staurozoa and possesses an unusually large holdfast disc, we are not in a position to assign it to the class Staurozoa on the basis of available evidence. Cubozoans can also possess bifurcating tentacles and fourfold symmetry, but extant forms are pelagic, not benthic as inferred for *Haootia*.

Interestingly, symplesiomorphies within the Medusozoa have been proposed to include the presence of four intramesogleal muscles [40]. The Medusozoa are usually considered to have a long evolutionary history, with divergence from the Octocorallia conservatively estimated to have taken place at least approximately 571 Ma [86]. If correct, medusozoan ancestors, and indeed diverse cnidarian ancestors, would be expected within Late Ediacaran marine environments. The suggestion that Staurozoa is the sister group to all other medusozoan classes ([40,87], though see [58]) potentially indicates a similarly ancient evolutionary history for that clade. Further comparisons with the body plans of extant cnidarians are limited by our poor understanding of deep sea forms [88], and the absence of many extinct forms (cf. [59]). Until further morphological evidence is obtained, we therefore suggest that the muscular *H. quadriformis* n. gen., n. sp. occupied a position within the Cnidaria, and potentially within the stem-group Medusozoa.

## 5. The significance of a cnidarian at approximately 560 ma

Interpretation of *H. quadriformis* as a muscular cnidarian leads us to examine the early fossil record of the phylum Cnidaria. Cnidarians appear to have diversified into several major clades by the Middle Cambrian, as evidenced by the presence of probable anthozoan actinians [89–92] and corals [93–96], scyphozoans [97], possible hydrozoans and cubozoans [66,98] and cnidarians of unknown affinity [99] in Lower and Middle Cambrian strata, with conulariids [100] and mass strandings of medusae [101,102] additionally reported in the Upper Cambrian (see also [71]). Some of the earliest interpretations of the original Ediacara biota of Australia proposed cnidarian medusoid affinities for discoidal specimens [103–105], but many of these have since been disputed (e.g. [71,106]). Similarly, interpretation of *Inaria* as an actinian-grade, muscle-bearing polyp [107] has been questioned following taphonomic and morphodynamic analysis [77]. Other reports of cnidarians in latest Ediacaran rocks include *Pambikalbae* as a ?hydrozoan [108]; interpretation of the tubular fossils *Corumbella* and *Vendoconularia* as scyphozoans similar to the conulariids [9,11,109]; discussion of the biomineralized genera *Cloudina* and *Namacalathus* as ‘cnidariomorphs’ [110]; and the possible calcified cnidarian *Namapoikia* [111]. Fossils from the Late Ediacaran Doushantuo Formation have been tentatively compared to tabulates [112,113] and hydrozoans [10]. Elsewhere, the recent reinterpretation of certain Middle Ediacaran carbonaceous fossils from the Lantian Biota as potential conulariids [114] is of interest. Traces of actinian-like locomotion in deep marine sediments approximately 565–560 Ma are also germane here [22,23]. All claims for Neoproterozoic metazoans should be critically assessed on a case-by-case basis, much as with the early sponge fossil record [8]. At the time of writing, however, the studies cited above clearly indicate morphological diversity of fossil cnidarian candidates in the Late Ediacaran/Early Cambrian. Such fossils have also been used to help calibrate recent molecular estimates of bilaterian–cnidarian divergence during the Ediacaran Period [33].

Cnidarian-like body fossils from Newfoundland at approximately 560 Ma also raise important questions about tissue differentiation, feeding strategy, food sources and the complexity of Late Ediacaran ecosystems. Our interpretation of *H. quadriformis* as a muscular metazoan of cnidarian grade arguably represents the earliest known evidence for preservation of muscular tissue in the geological record, and one of the earliest claims for a eumetazoan (*see also* [10,114]). *Haootia* therefore delivers a key calibration point for studies of early Eumetazoan evolution and body symmetry.

## Supplementary Material

Electronic Supplementary Material
