# Supplementary material for: Haootia quadriformis n. gen., n. sp., interpreted as a muscular cnidarian impression from the Late Ediacaran period (approx. 560 Ma)
Source: Proc Biol Sci. 2014 Oct 22;281(1793):20141202. doi: 10.1098/rspb.2014.1202 (PMC4173675; doi:10.1098/rspb.2014.1202)
Supplement: Electronic Supplementary Material [file rspb20141202supp1.pdf]

Haootia quadriformis n. gen., n. sp., interpreted as a muscular cnidarian impression from the late Ediacaran Period (~560 Ma)

ELECTRONIC SUPPLEMENTARY MATERIAL

Liu, A.G., Matthews, J.J., Menon, L.R., McIlroy, D. and Brasier, M.D.

Supplementary Text S1

Sedimentology and Paleontology of the Back Cove fossil locality

The *Haootia quadriformis* n. gen., n. sp. holotype specimen was first found by one of us (MDB) on a bedding plane in Back Cove, in the vicinity of the town of Melrose, on the Bonavista Peninsula of Newfoundland (Fig. S1). Late Ediacaran rocks of the Conception and St. John's Groups occur here as an anticline commonly referred to as the Catalina Dome [115]. Fossilized Ediacaran macro-organisms described from the Catalina Dome show comparable taxonomic diversity, abundance, and taphonomic fidelity to both the contemporaneous Mistaken Point biota of the southern Avalon Peninsula ~200 km to the south [37], and the Mercian Assemblage from Charnwood Forest, U.K. [116]. Assemblages are dominated by frondose rangeomorph taxa (cf. refs 33, 38, 117). The holotype *Haootia* specimen locally lies within a succession of thin- to medium-bedded, fining-upwards cycles of fine sandstones and mudstones (Fig. S2), attributed to the lower Fermeuse Formation (St. John's Group). Ripple-cross-lamination in some of the sands, several erosional surfaces, and normally-graded bedding are consistent with the sedimentary packages recording distal turbidity currents or density flows deposited on a slope (as inferred by numerous slump deposits within the surrounding Fermeuse Formation), in a fore-arc basin setting [118].

Around 50% of the beds in the vicinity of the site are capped by a band of medium to coarse-grained brown volcaniclastic material, 1–5 mm thick (Fig. S2D, arrowed).

Haootia quadriformis n. gen. n. sp. lies on a ~ 52 mm-thick fine siltstone, the top 7.5 mm of which is a texturally-mottled hemi-pelagite (cf. refs 44, 119; Fig. S2E). The fossiliferous surface is capped by 6 mm of fine-sand-sized buff-weathering normally-graded tuff. Secondary pyrite crystals roughly 1 mm in diameter are present within this tuff, and its angular clasts are set within a strongly altered matrix. Soft tissues are considered to be preserved via early diagenetic replication of tissue morphology by framboidal pyrite (cf. ref. 77). The fossiliferous surface is cut by three primary cleavage directions, none of which are related to the trends of the linear fibers comprising the fossils described herein.

Other recognizable Ediacaran genera on the bedding surface include *Charniodiscus* (Fig. S3B–C), *Bradgatia*, ?*Primocandelabrum* (Fig. S3A), *Hiemalora*, *Vinlandia*, several ivesheadiomorphs, and concentrically-banded '*Spriggia*-morphs' of *Aspidella terranovica* (cf. ref. 120; Fig. S3D–F; likely to represent holdfast structures of frondose organisms). All fossils are of low (<2 mm) topographic relief, and are observed on the top surface of the bed. Morphological details down to 0.5 mm in resolution are preserved, with taphonomic fidelity of the discoidal fossils unusually being better than that of associated rangeomorph branching (Fig. S3).

No accurate radiometric dates have yet been published for successions from the Bonavista Peninsula, but the late Ediacaran units have been lithostratigraphically correlated with those of the Conception and St. John's Groups from the Avalon Peninsula [37] (Fig. S1). If those correlations are correct, the widely cited date of 565±3 Ma obtained by U-Pb dating of zircons within a volcanic tuff from the Mistaken Point Formation (published without

supporting isochrons in an abstract, ref. 24) would suggest that both the Back Cove locality and the Burnt Point paratype locality are younger than 565 Ma (Fig. S1).

Supplementary Text S2

Cnidarian biology and musculature

The Phylum Cnidaria is united by the presence of cnidocysts, and the use of either or both of two body states; a benthic polyp, and a free-swimming medusa. Other important morphological characteristics are the presence of a nerve net; a single body cavity with a single opening; tentacles; and either a primary radial symmetry about an oral/aboral axis, or bi-radial symmetry with retention of external radial features [73]. Traditionally cnidarians have been divided into two main groups - the Anthozoa (including the Hexacorallia and Octocorallia), and the Medusozoa (Cubozoa, Hydrozoa, Scyphozoa and Staurozoa) - but the phylogenetic relationships within and between these groups (e.g. refs 55, 83, 84, 121-123), are in flux owing to the ongoing generation of genomic and genetic data.

Extant Cnidarians can possess both smooth and striated muscular tissue [60, 124], with the arrangement and abundance of such tissues varying widely amongst the phylum [125]. Striated muscle is typically found in the medusa stage of a taxon, where it is most commonly located in the bell and is used to power locomotion through the water column. In contrast, the polyp and larval stages are generally composed of epithelial or sub-epithelial smooth muscle (ref. 61 and references therein). Both smooth and striated muscle cells are composed of myofibrils comprising filamentous actin and myosin proteins (e.g. ref. 126).

Whereas the actin and myosin filaments are relatively poorly arranged in smooth muscle, in striated muscle they form well-organized units known as sarcomeres, which are arranged in regular arrays along the myofibrils [127, 128]. Myofibrils themselves are then encased in a collagenous membrane and arranged in bundles into muscle fibers. Muscular tissues are further divided into true muscle fibers (myocytes), and epithelial muscle, the latter considered the most primitive type of contractile tissue [129]. Both variants can be found within members of the Cnidaria [127]. Actins and myosins are considered to have a long evolutionary history, and have even been postulated to have been present in the last eukaryotic common ancestor [130, 131]. Interestingly, contractile filaments (not considered to represent true muscle) have been recognized in several groups of protists, including the heliozoans, and the ciliates (such as the taxa *Vorticella*, *Stentor,* and *Zoothamnium*; ref. 73, p. 47).

Mesoderm-like structures have been documented across the extant cnidarian tree (reviewed in ref. 60). In most medusae, the striated cells are located sub-epidermally (Krasinska in ref. 60), but other workers consider all cnidarian musculature to be epithelial musculature, and ectodermal in origin [132], thus questioning whether these structures truly represent a third germ layer. It has also been suggested that striated musculature would necessarily have evolved in tandem with the nervous and digestive systems, in order to produce a functional digestive cavity that is surrounded by musculature [61].

On the basis of molecular and ontogenetic studies, it has been argued that cnidarians, bilaterians, and ctenophores (which can also possess striated muscle in their tentacles [61] and have previously been proposed to be triploblastic (e.g. refs 133, 134), would have all originated from a common ancestor that was motile, triploblastic, and in possession of striated muscle tissue [61]. However, there are subtle differences between striated muscle in

cnidarians and bilaterians [132], and recent genetic research has demonstrated that muscle in these clades is constructed using entirely different sets of genes and proteins [135], arguing for convergent evolution of such tissues. The structure of the cnidarian-bilaterian common ancestor therefore remains unresolved, but musculature being a primitive eumetazoan character is a plausible possibility. Proposed muscular tissue in the late Ediacaran macrofossil *Kimberella* (ref. 14, fig. 18) displays fine symmetrical transverse wrinkles, but we do not consider *H. quadriformis* to possess any close phylogenetic relationship with this taxon. No other Ediacaran megafossil has been claimed to document preserved muscular tissue.

References only in the Supplementary Material

115. O'Brien S.J., King A.F. 2004 Ediacaran fossils from the Bonavista Peninsula (Avalon zone), Newfoundland: Preliminary descriptions and implications for regional correlation. *Current Research, Newfoundland Department of Mines and Energy Geological Survey* **04-1**, 203-212.

116. Wilby P.R., Carney J.N., Howe M.P.A. 2011 A rich Ediacaran assemblage from eastern Avalonia: Evidence of early widespread diversity in the deep ocean. *Geology* **39**(7), 655-658.

117. Narbonne G.M. 2004 Modular construction in the Ediacaran biota. *Science* **305**, 1141-1144.

118. Mason S.J., Narbonne G.M., Dalrymple R.W., O'Brien S.J. 2013 Paleoenvironmental analysis of Ediacaran strata in the Catalina Dome, Bonavista Peninsula, Newfoundland. *Canadian Journal of Earth Sciences* **50**, 197-212.

119. Brasier M.D., Antcliffe J.B., Bright M., Liu A.G., Matthews J.J., McIlroy D., Wacey D. In Preparation Extreme sulfur cycling within the earliest deep-sea macrobiotas.

120. Gehling J.G., Narbonne G.M., Anderson M.M. 2000 The first named Ediacaran body fossil, *Aspidella terranovica*. *Palaeontology* **43**(3), 427-456.

121. Bayha K.M., Dawson M.N., Collins A.G., Barbeitos M.S., Haddock S.H.D. 2010 Evolutionary relationships among Scyphozoan jellyfish families based on complete taxon sampling and phylogenetic analyses of 18S and 28S ribosomal DNA. *Integrative and Comparative Biology* **50**(3), 436-455.

122. Bentlage B., Cartwright P., Yanagihara A.A., Lewis C., Richards G.S., Collins A.G. 2010 Evolution of box jellyfish (Cnidaria: Cubozoa), a group of highly toxic invertebrates. *Proceedings of the Royal Society, London B* **277**, 493-501.

123. Cartwright P., Nawrocki A.M. 2010 Character evolution in Hydrozoa (phylum Cnidaria). *Integrative and Comparative Biology* **50**(3), 456-472.

124. Calgren O. 1949 A survey of the Ptychodactiaria, Corallomorpharia and Actinaria. *KVA Handl* **1**, 1-121.

125. Schmid V. 1988 The potential for transdifferentiation and regeneration of isolated striated muscle of medusae in vitro. *Cell Differentiation* **22**, 173-182.

126. Hejnol A. 2012 Muscle's dual origins. *Nature* **487**, 181-182.

127. Chiodin M., Achatz J.G., Wanninger A., Martinez P. 2011 Molecular architecture of muscles in an acoel and its evolutionary implications. *Journal of Experimental Zoology Part B (Molecular and Developmental Evolution)* **316**, 427-439.

128. Sparrow J.C., Schock F. 2009 The initial steps of myofibril assembly: integrins pave the way. *Nature Reviews* **10**, 293-298.

129. Reiger R.M., Ladurner P. 2003 The significance of muscle cells for the origin of mesoderm in Bilateria. *Integrated Comparitive Biology* **43**, 47-54.

130. Foth B.J., Goedecke M.C., Soldati D. 2006 New insights into myosin evolution and classification. *Proceedings of the National Academy of Sciences, USA* **103**(10), 3681-3686.

131. Richards T.A., Cavalier-Smith T. 2005 Myosin domain evolution and the primary divergence of eukaryotes. *Nature* **436**, 1113-1118.

132. Burton P.M. 2008 Insights from diploblasts; the evolution of mesoderm and muscle. *Journal of Experimental Zoology (Mol Dev Evol)* **310B**, 5-14.

133. Hernandez-Nicaise M.-L., Franc J.M. 1993 Embranchment des ctenaires: Morphologie, Biologie, Ecologie. In *Traite de Zoologie Cnidaires, Ctenaires* (ed. Grasse P.-P.), pp. 943-1055. Paris, Masson.

134. Martindale M.Q., Henry J.Q. 1999 Intracellular fate mapping in a basal metazoan, the ctenophore *Mnemiopsis leidyi*, reveals the origins of mesoderm and the existence of intermediate cell lineages. *Developmental Biology* **246**, 243-257.

135. Steinmetz P.R.H., Kraus J.E.M., Larroux C., Hammel J.U., Amon-Hassenzahl A., Houliston E., Worheide G., Degnan B.M., Technau U. 2012 Independent evolution of striated muscles in cnidarians and bilaterians. *Nature* **487**, 231-234.

136. Schmid V. 1969 Zur gametogenese von *Podocoryne carnea*. *M Sars Rev Suisse Zool* **76**, 1071-1078.

SUPPLEMENTARY FIGURES

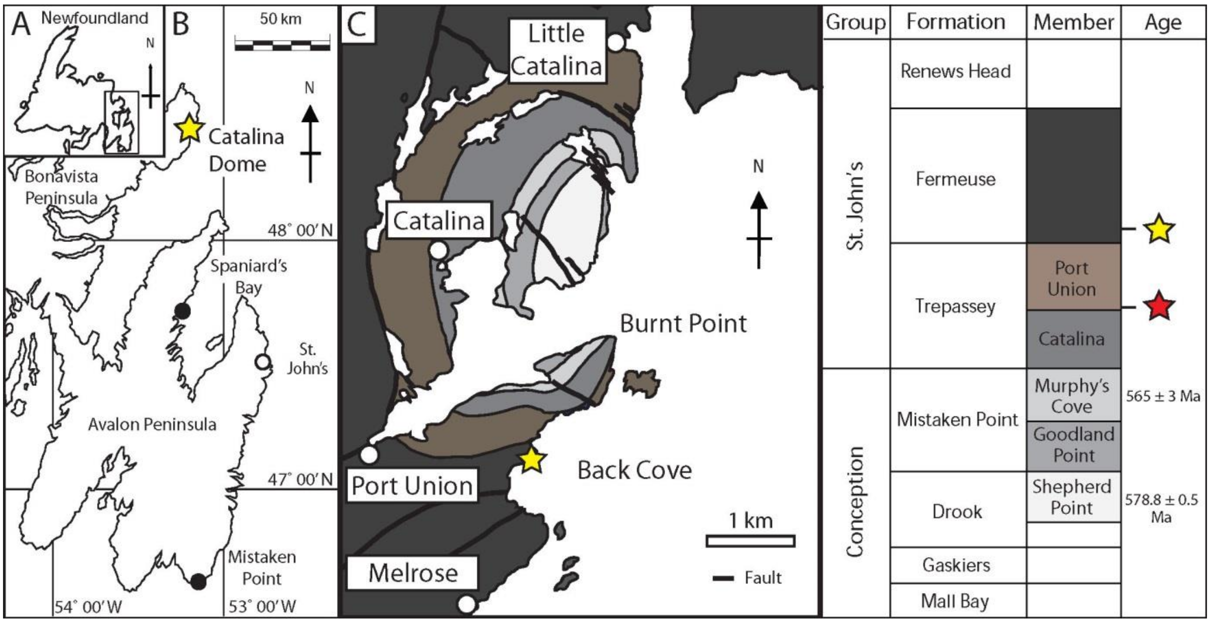

Supplementary Figure S1. Location and stratigraphic position of the *Haootia quadriformis* n. gen., n. sp. holotype locality; Back Cove, Catalina Dome, Bonavista Peninsula, Newfoundland. (**A)** Outline map of Newfoundland, showing the Avalon and Bonvista Peninsulas in box. (**B)** The Avalon and Bonavista Peninsulas, showing locations of the Catalina Dome (yellow star) and other major Ediacaran fossil sites (black circles). (**C)** Geological map of the Catalina Dome, redrawn after [37], showing settlements and the holotype locality in Back Cove (yellow star). Key to the geological units can be found in the stratigraphic column, which follows [37]. Radiometric dates are taken from lithostratigraphically correlated units on the Avalon Peninsula [24, 34], though note neither cited study presents an isochron to permit scrutiny of their dates. Red star indicates the level from which the *H. quadriformis* paratype originates.

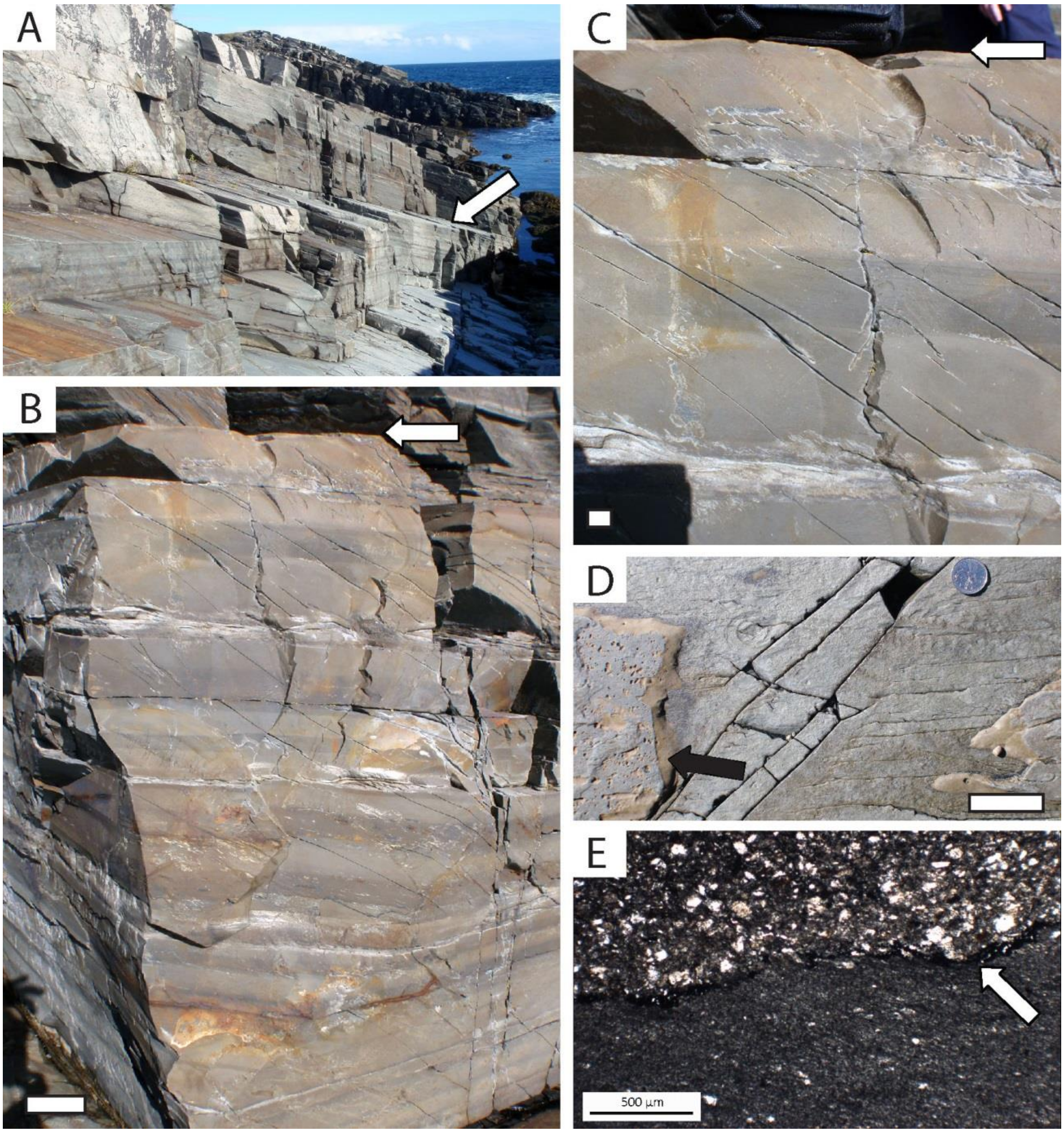

Supplementary Figure S2. The sedimentology of the *Haootia quadriformis* n. gen., n. sp. holotype bedding plane at Back Cove, Catalina Dome, Newfoundland. White arrows indicate the level at which the fossils are found. (**A)** View of the locality. (**B)** Thin–medium bedded turbidites of the lower Fermeuse Formation beneath the fossil bed. (**C)** Close-up view of the sediments directly beneath the fossil surface. (**D)** Plan view of the bedding plane, showing the brown sandy tuff that covered the surface (black arrow). This tuff often contains cubes of rusty iron oxides, replacing secondary euhedral pyrite. A *Charniodiscus* is also present in this image (frond lies beneath the coin). (**E)** The sedimentology of the sediment-tuff interface

upon which the fossils are found, viewed under crossed-polars. The surface between the fine sand-silt under-bed and the coarser feldspar-rich over-bed is coated by a thin drape of spheroidal iron oxides and iron staining, interpreted to be oxidised replacements of pyrite framboids. Scale bar in C = 10 mm, B, D = 50 mm.

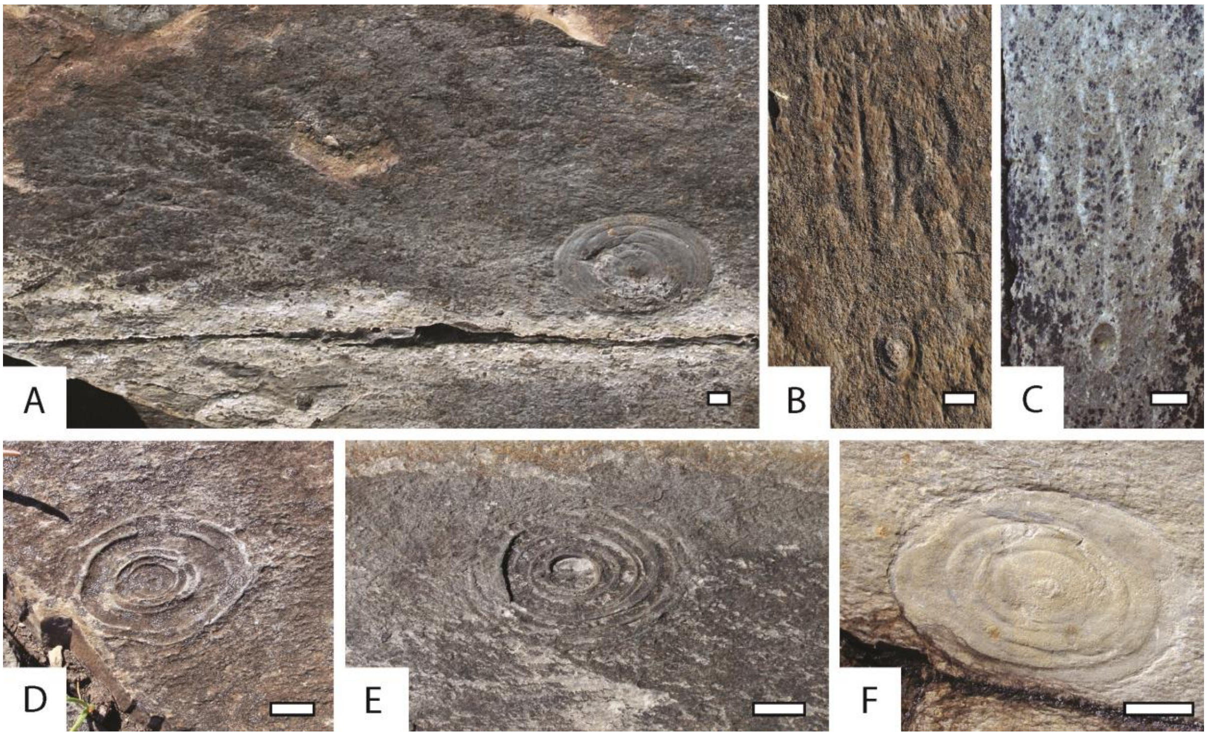

Supplementary Figure S3. Typical taxa of the late Ediacaran Avalonian biota, from the Back Cove bedding plane yielding the *H. quadriformis* n. gen., n. sp. holotype. (**A)** *Primocandelabrum* sp. (cf. ref. 37), with an extremely finely banded concentric disc, but poor preservation of rangeomorph branching. (**B–C)** *Charniodiscus* sp. (cf. ref. 37). (**D–F)** *Aspidella terranovica* (cf. ref. 120) with prominent concentric banding; these are interpreted to represent collapsed holdfast structures of frondose taxa. All scale bars = 10mm.

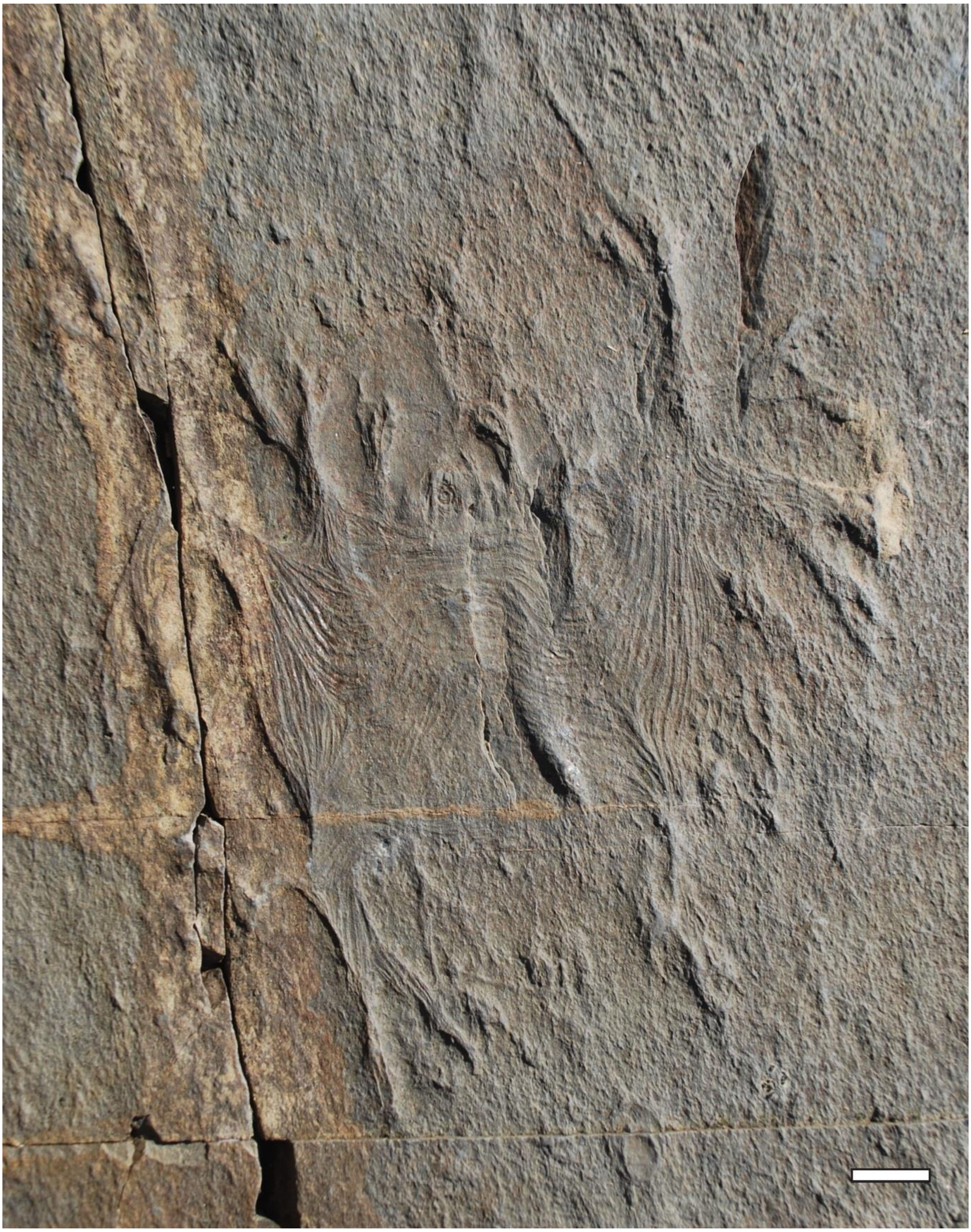

Supplementary Figure S4. The holotype specimen of *Haootia quadriformis* n. gen., n. sp., from Back Cove, Bonavista Peninsula, Newfoundland. A plaster replica (the plastotype) of this specimen resides in the Oxford University Museum of Natural History: OUM ÁT.424/p. This image is a larger version of that used in Fig. 1A. Scale bar = 10mm.

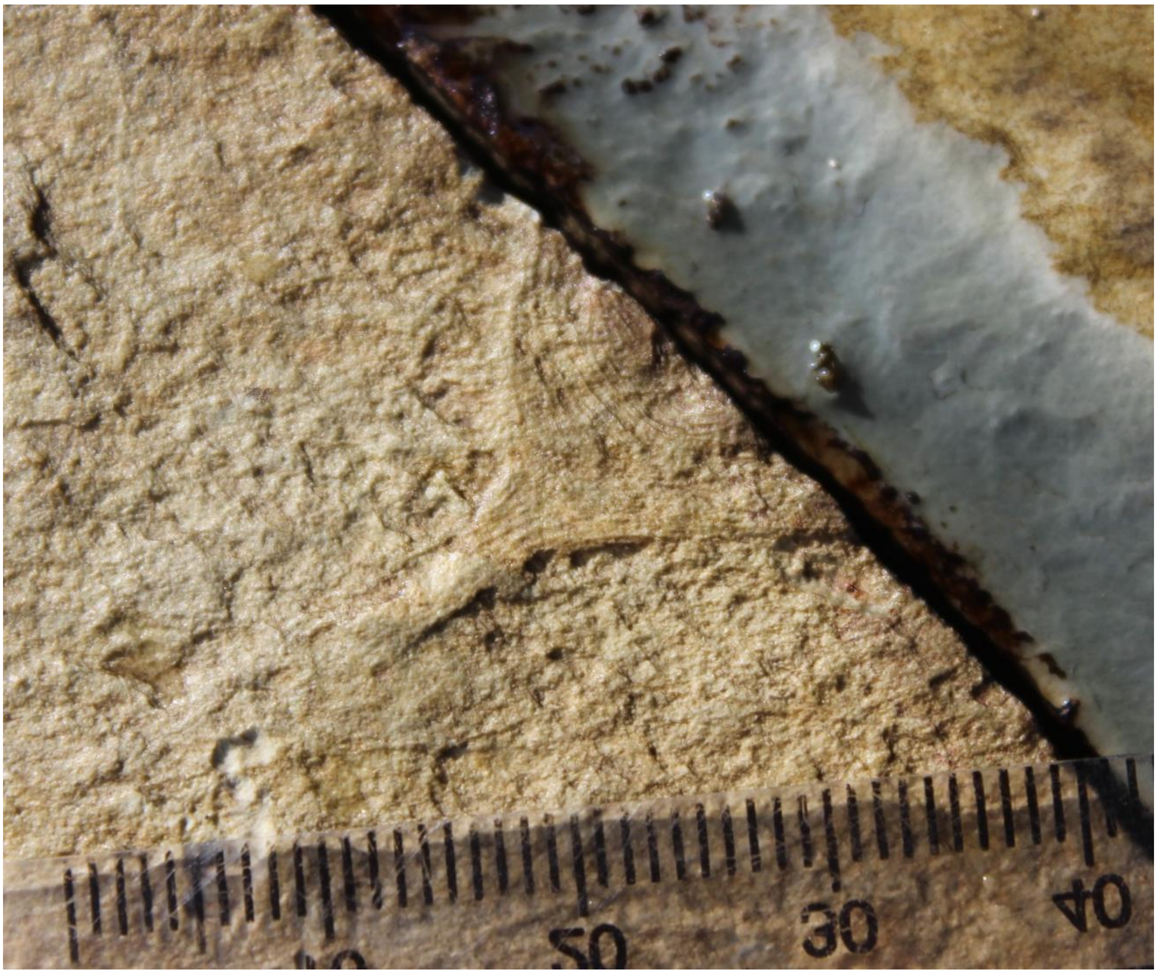

Supplementary Figure S5. The paratype of *Haootia quadriformis* n. gen., n. sp., from the Trepassey Formation of Burnt Point, Bonavista Peninsula, Newfoundland. This incomplete specimen remains uncollected in the field. Scale bar gradations are in millimeters.

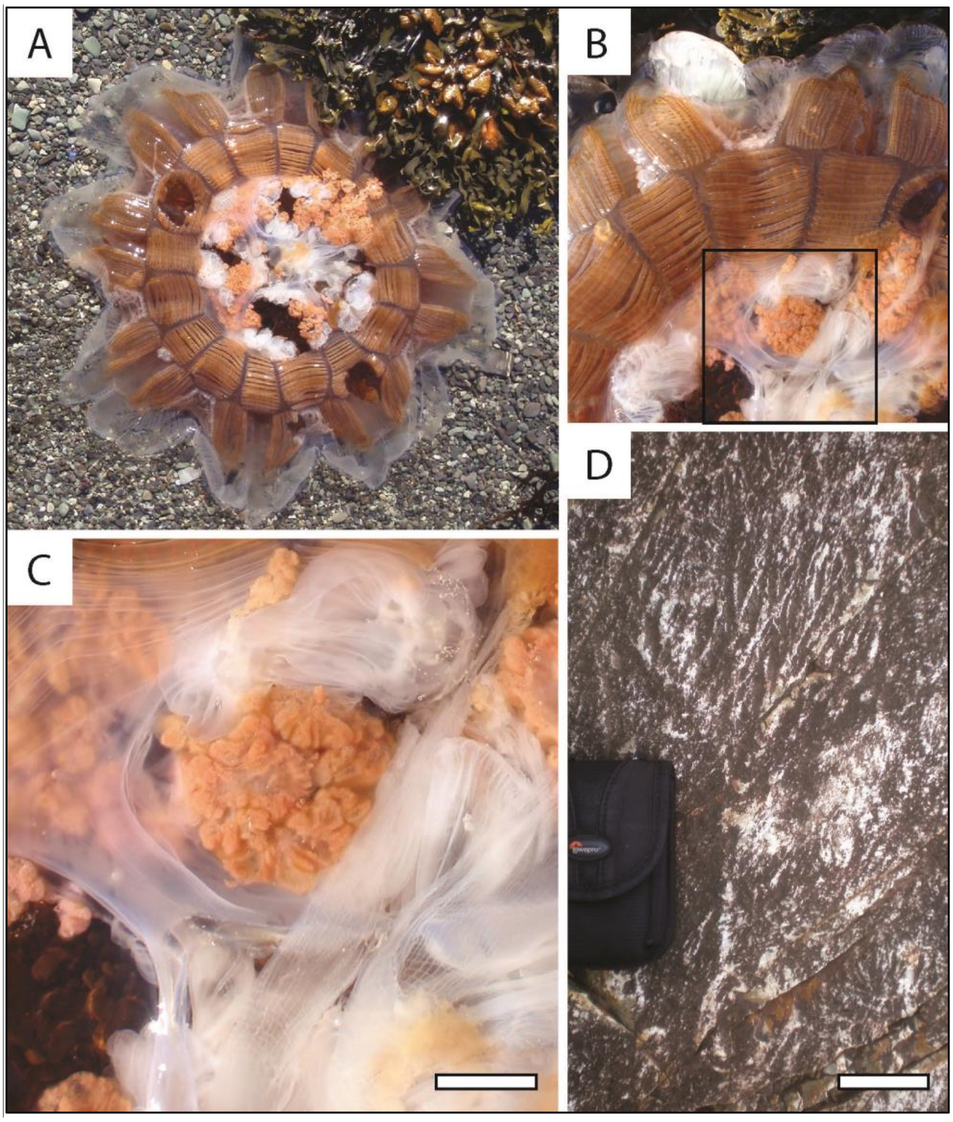

Supplementary Figure S6. Organisms to which *Haootia quadriformis* n. gen., n. sp. can be compared. **(A–C)** The extant “Lion’s Mane” jellyfish *Cyanea capillata*, showing increasingly finer detail of musculature on the underside of the bell and in the manubrium. (**D)** *Primocandelabrum* sp., a late Ediacaran rangeomorph from the Bonavista Peninsula, Newfoundland. Phanerozoic observations demonstrate that medusae are generally not preserved in deep seafloor environments, likely because small individuals break down rapidly, while larger specimens remain buoyant in the water column; strandings or very shallow and calm environments are considered to be more suitable for medusa preservation [61, 136]. Scale bar in C = 10 mm, D = 50 mm.
